# Studies related to osteosarcoma and metabolism from 1990 to 2022: A visual analysis and bibliometric study

**DOI:** 10.3389/fendo.2023.1144747

**Published:** 2023-03-06

**Authors:** Zhuce Shao, Shuxiong Bi

**Affiliations:** Third Hospital of Shanxi Medical University, Shanxi Bethune Hospital, Shanxi Academy of Medical Sciences, Tongji Shanxi Hospital, Taiyuan, China

**Keywords:** osteosarcoma, metabolism, bibliometrics, visualized study, trends

## Abstract

**Background:**

Osteosarcoma is the most common primary bone tumor, its high incidence of metastasis and poor prognosis have led to a great deal of concern for osteosarcoma. In many cancer types, metabolic processes are important for tumor growth progression, so interfering with the metabolic processes of osteosarcoma may be a therapeutic option to stall osteosarcoma progression. A key mechanism of how metabolic processes contribute to the growth and survival of various cancers, including osteosarcoma, is their ability to support tumor cell metabolism. Research related to this field is a direction of great importance and potential. However, to our knowledge, no bibliometric studies related to this field have been published, and we will fill this research gap.

**Methods:**

Publications were retrieved on January 1, 2023 from the 1990-2022 Science Citation Index of the Web of Science Core Collection. The Bibliometrix package in R software, VOSviewer and CiteSpace software were used to analyze our research directions and to visualize global trends and hotspots in osteosarcoma and metabolism related research.

**Results:**

Based on the search strategy, 833 articles were finally filtered. In this area of research related to osteosarcoma metabolism, we found that China, the United States and Japan are the top 3 countries in terms of number of articles published, and the journals and institutions that have published the most research in this area are Journal of bone and mineral research, Shanghai Jiao Tong University. In addition, Baldini, Nicola, Reddy, Gs and Avnet, Sofia are the top three authors in terms of number of articles published in studies related to this field. The most popular keywords related to the field in the last 30 years are “metabolism” and “expression”, which will guide the possible future directions of the field.

**Conclusion:**

We used Bibliometrix, VOSviewer, and Citespace to visualize and bibliometrically analyze the current status and possible future hotspots of research in the field of osteosarcoma metabolism. Possible future hotspots in this field may focus on the related terms “metabolism”, “expression”, and “migraation”.

## Introduction

Abnormal metabolism is the main feature of cancer. Abnormal cancer metabolism, changes in the anabolic pathways of certain substances such as lipid metabolism, glutamine metabolism and glucose metabolism plays a crucial role in tumorigenesis, development and metastasis (1–6).The abnormal metabolism of cancer cells is the result of genetic mutations, and more importantly, it can also directly affect the signaling of tumor cells and the responses made by the final cells (7). In recent years there has been an increasing interest in the field of cancer metabolism in many basic experiments and clinical trials. Researchers are aiming to determine whether cancer development or progression can be halted by curbing the metabolic changes in cancer. Metabolism is also important in the development, progression and metastasis of osteosarcoma.

Osteosarcoma (OS) is the most common malignant bone tumor, occurring in children and adolescents, with an incidence of less than 5 per million (8, 9). Although treatment for OS includes a variety of treatments, including surgical resection and chemotherapy, overall survival remains poor (10). One of the main reasons for the poor prognosis is that the metabolic process of osteosarcoma is not well understood, and the development, progression, or metastasis of osteosarcoma is not well prevented. development or metastasis.

Bibliometrics is an emerging approach in recent years to provide an in-depth scientific qualitative and quantitative analysis and visualization of the published literature in a specific research area, which can provide a better and clearer understanding of the current status and future trends of a research area (11, 12).Unlike previous traditional systematic assessments, bibliometric analyses focus on author collaboration networks and national regions of the literature, and on the interconnectedness of different research institutions and individual journals publishing in the field (13).To the best of our knowledge, there are no bibliometric studies on the metabolism-related aspects of osteosarcoma research to date. Bibliometrics has been used in several disciplines, such as digestive (14), neurological (15), and cancer systems (16). Therefore, the purpose of this study was to provide an in-depth analysis and visualization of research on osteosarcoma metabolism from 1990 to 2022, as well as to assess the current state of research and future trends and hot spots in this area of osteosarcoma metabolism.

## Methods and materials

### Data collection and retrieval process

**Figure 1:**
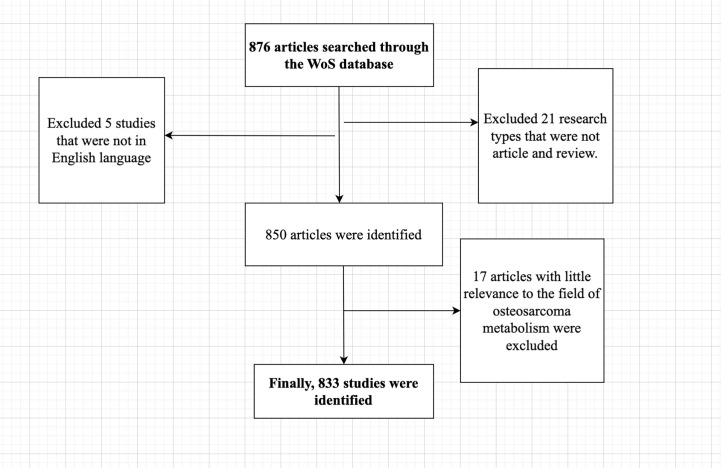
Diagram of the process of screening literature.

We searched the Web of Science (WoS) systematically for the period from January 1, 1990 to December 20, 2022. The WoS contains a huge number of journal categories and is the most frequently read database (17).Compared to other databases, the WoS database is more comprehensive, clearer, and broader (18),and is the most suitable database for bibliometric research (19–21).We also downloaded the data on January 1, 2023. Search terms included: TS = (osteosarcoma) and TS = (metabolism). After careful and multiple screening, only theses and review articles were retained, we removed literature that was not relevant to our study topic and restricted the language type to English only, extracted and saved in txt format, where these files were plain text files with full records and cited references retained for better visualization and bibliometric analysis of our study. **Figure 1** can better demonstrate our process of screening the literature.

The study did not require the consent of the Ethical Medical Council.

### Presentation of the software and its tools for visualization and bibliometrics to be used in this study

VOSviewer (22)and CiteSpace (23) can display the collaborative network relationships and keywords between different authors, countries, and institutions of the published literature in a certain research area. In the results presented by the CiteSpace software, each point in the graph represents an element, which can be an author, a country, an institution or a keyword. The lines between the points represent the strength of their relevance or the frequency of collaboration and relationships (23, 24). The sets of elements in different kinds of colors indicate different clusters (25).

In addition, R language software (26) is required, of which the Bibliometrix R package provides a quantitative tool for bibliometric analysis studies.

## Results

### Information on published literature on research in this field of osteosarcoma metabolism

From the results of integrating all the published literature collected in the field of osteosarcoma metabolism research, we found a total of 833 studies, 404 journals, 5,032 authors shooting research in the field of osteosarcoma metabolism over a 30-year period from 1990 to 2022, after screening, and an additional 1,164 institutions and 56 countries were counted. A total of 37,370 articles from 4,232 journals were cited in publications in the field of osteosarcoma metabolism.

### Analysis of the number of articles published in the field of osteosarcoma metabolism by country and region

Fifty-six countries have contributed to the study of Ewing sarcoma, including 261 studies from China and 207 from the United States, whose average citation rate is 48.28. The top 3-5 countries publishing literature related to this field are Japan, Italy and Germany. Canada has only 33 published papers, but Canada leads in terms of the average number of citations with 78.94, which may also indicate the higher recognition of Canadian papers. The 10 countries with the highest number of publications in this area of osteosarcoma metabolism are listed in **Table 1**.

**Table 1:** Top 10 countries with the highest number of articles published in the field of osteosarcoma metabolism.

Rank	Country	Documents	Citations	Average Citation/Publication
1	China	261	3994	15.30
2	Usa	209	10090	48.28
3	Japan	66	2313	35.05
4	Italy	63	1776	28.19
5	Germany	49	1642	33.51
6	England	44	2286	51.95
7	Canada	33	2605	78.94
8	France	33	2461	74.58
9	Spain	20	454	22.70
10	Netherlands	19	1404	73.89

**Figure 2:**
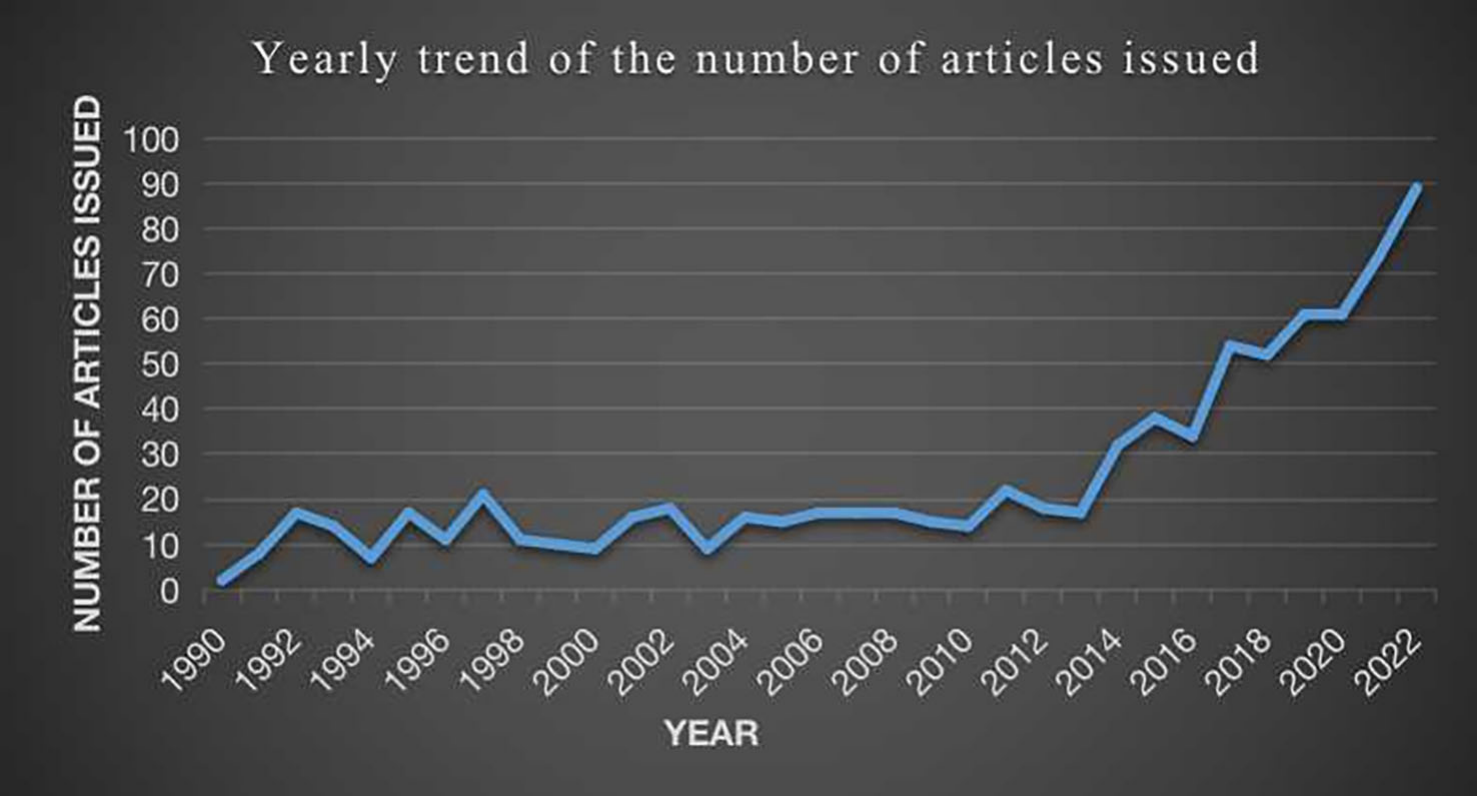
Trends in the number of articles published over time in the field of osteosarcoma metabolism.

In addition, as can be seen from the line graph in **Figure 2**, the volume of papers in the field of osteosarcoma metabolism in the cluster with the trend line shows an overall steady upward trend in the volume of papers in the field related to osteosarcoma metabolism.

### Number of articles published in different journals in the field of osteosarcoma metabolism

**Figure 3:**
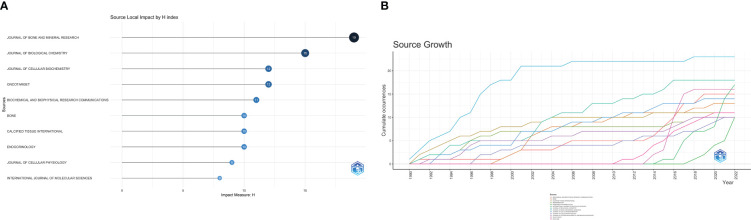
Trends and H-index of the top 10 journals in terms of number of articles published. **(A, B)** shows the H-index of the journals in this field, and the trend of the number of articles published in the journals in this field over time, respectively.

According to the analysis of the field of osteosarcoma metabolism research by VOSViewer software, it was found that “Journal of bone and mineral research” was the most published journal in this field with 23 publications and a total of 1715 citations. In addition, the journal Bone had the highest average number of citations with 93.54, indicating that the average quality of publications in this field in this journal is high. We found that these journals are mostly related to bone, tumor or endocrine and metabolism. With an impact factor of 6.390, the journal “ Journal of bone and mineral research “ is an excellent Journal Citation Reports (JCR) Division 1 journal with a high rating in the industry. **Table 2** shows the top 10 journals in terms of the number of articles published on osteosarcoma metabolism-related research content. In addition, **Figure 3A** shows the H-index of the top 10 journals in the field of osteosarcoma metabolism in terms of the number of published articles between 1990 and 2022, and **Figure 3B** shows very clearly the trend of the top journals in terms of the number of articles published in the field over the recent years.

**Table 2:** Top 10 journals published in the field of osteosarcoma metabolism.

Rank	Journals	Documents	Citations	Average Citation/Publication
1	Journal of bone and mineral research	23	1715	74.57
2	Journal of biological chemistry	18	1554	86.33
3	International journal of molecular sciences	17	142	8.35
4	Oncotarget	16	544	34.00
5	Biochemical and biophysical research communications	15	504	33.60
6	Journal of cellular biochemistry	14	447	31.93
7	Bone	13	1216	93.54
8	Calcified tissue international	11	450	40.91
9	Plos one	11	298	27.09
10	Endocrinology	10	409	40.90

### Analysis of the authors of published literature in the field of osteosarcoma metabolism

According to Price Law, m=0.749*√n_max_=2.247(n_max_=9), then authors with more than or equal to 3 publications are defined as core authors in this field, with 101 individuals, and the details of the top 10 authors in terms of number of publications are shown in **Table 3**. Among them, we found that the studies published by Baldini, Nicola, the number one author in the field of osteosarcoma metabolism, are very well fitted to analyze the role and impact of multiple substances such as stem cells or acidosis in osteosarcoma metabolism (27, 28). He has published a total of nine papers related to the field, with a total of 248 citations and an average citation count of 27.56. In addition, Reddy, Gs, who tied for first place in terms of number of publications, focused on the relationship between osteosarcoma and vitamin metabolism (29).

**Table 3:** The top 10 authors in terms of number of publications.

Rank	Author	Documents	Citations	Average Citation/Publication
1	Baldini, nicola	9	248	27.56
2	Reddy, Gs	9	308	34.22
3	Avnet, Sofia	8	229	28.63
4	Hall, ih	6	135	22.50
5	Jones, G	6	118	19.67
6	Shang, peng	6	72	12.00
7	Hua Yingqi	5	113	22.60
8	Kubodera, n	5	206	41.20
9	Makin, Hlj	5	99	19.80
10	Okano, T	5	206	41.20

### Analysis of the different bodies of published literature in the field of osteosarcoma metabolism

The results presented by VOSviewer software show that Shanghai Jiao Tong University is the first in the world in terms of the number of publications in this field of research, with 28 articles on osteosarcoma metabolism and a total of 764 citations. In addition, University of Bologna and Zhejiang University ranked second and third, respectively, in the number of institutional publications in this field. **Table 4** shows information on the top 10 institutions worldwide in terms of the number of articles published in this field.

**Table 4:** Top 10 institutions with published articles in the field of osteosarcoma metabolism.

Rank	Organization	Documents	Citations	Total link strength
1	Shanghai jiao tong univ	28	764	19
2	Univ bologna	19	786	2
3	Zhejiang univ	17	346	4
4	Nanjing med univ	14	326	12
5	Brown univ	13	485	0
6	China med univ	13	249	10
7	Tongji univ	13	318	9
8	Cent south univ	11	85	7
9	Sun yat sen univ	11	177	1
10	Wuhan univ	11	119	2

### Collaborations between authors in the field of osteosarcoma metabolism from 1990 to 2022 were visualized and analyzed using VOSviewer software

**Figure 4:**
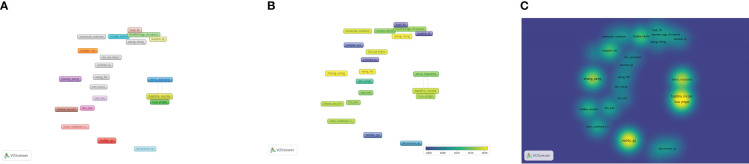
Collaboration chart of authors in the field (they have published more than 3 studies in the field). **(A-C)** are the connection network among authors, the graph of authors’ hotness over time, and the graph of authors’ density, respectively.

We used VOSviewer software to analyze the published literature on research related to osteosarcoma metabolism and found that a total of 5032 authors were involved in research on osteosarcoma metabolism, and we visualized the collaborative relationship graph of 101 authors who published more than 3 studies in this field, as shown in **Figure 4**. from **Figure 4**, it can be seen that in this field of research on osteosarcoma metabolism, the global collaborative This is one of the values of our study. With closer collaboration between researchers in this field on a global scale in the future, it is bound to give the field a better life and future value. **Figures 4A-C** are the connection network among authors, the graph of authors’ hotness over time, and the graph of authors’ density, respectively.

### A visual analysis of global institutional partnerships in the field of osteosarcoma metabolism from 1990 to 2022 was performed using VOSviewer software

**Figure 5:**
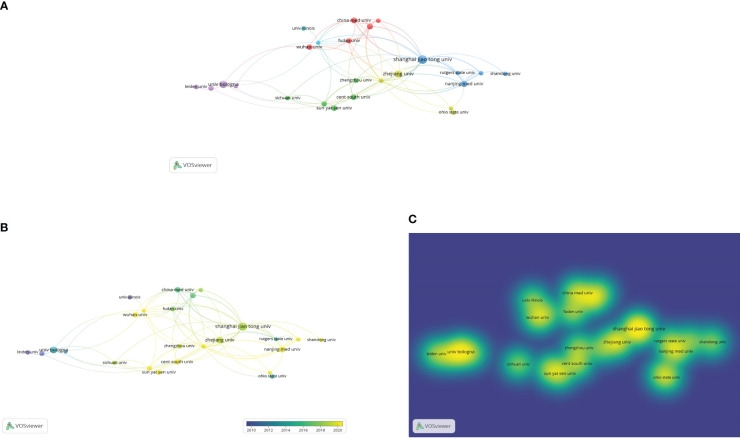
Global partnerships between institutions in the field of osteosarcoma metabolism. **(A-C)** are the network of connections between institutions, the graph of author’s hotness over time, and the graph of author’s density, respectively.

From **Table 4** in the previous paper, we have also found that Shanghai Jiao Tong University is the first in the number of articles published in this field, and **Figure 5** scientific research clearly shows that this institution has close cooperation with many other institutions. But it is more interesting to note that the top two institutions in this field do not have more obvious collaborative relationships, which is also worthy of our consideration. **Figures 5A-C** are the network of connections between institutions, the graph of author’s hotness over time, and the graph of author’s density, respectively.

### A visual analysis of global country and regional collaborations in the field of osteosarcoma metabolism from 1990 to 2022

**Figure 6:**
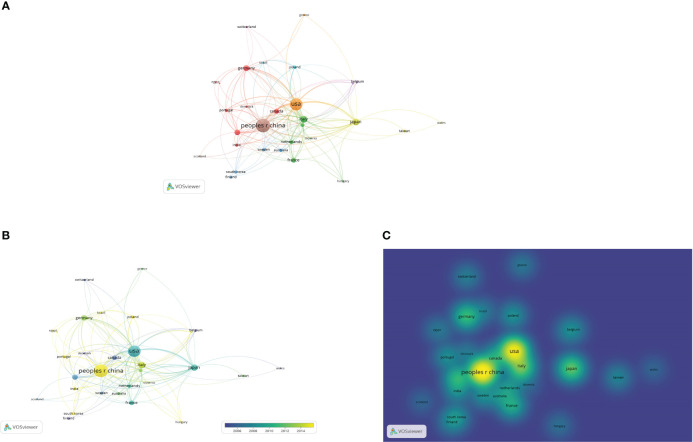
National and regional collaborations in the field of osteosarcoma metabolism. **(A-C)** are the network of connections between countries or regions, the graph of author’s hotness over time, and the graph of author’s density, respectively.

**Figure 7:**
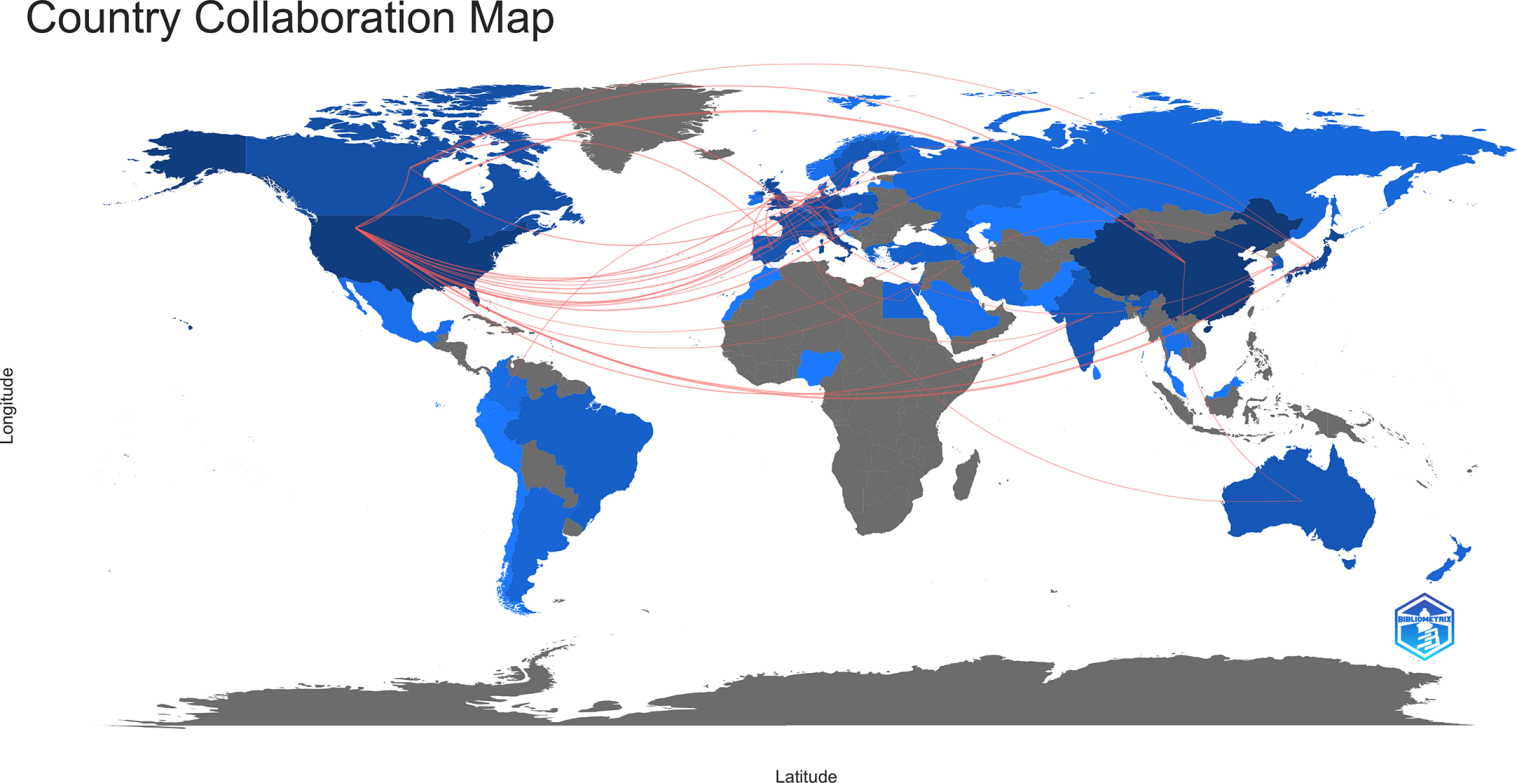
National and regional collaborations in the field of osteosarcoma metabolism as shown by the Bibliometrix package using R language software.

In the past 30 years, a total of 56 countries or regions worldwide have been involved in this research area of osteosarcoma metabolism, and it is clear from **Figure 6** that there is still close national cooperation in this area. A total of 30 countries or regions have published more than 5 relevant studies in this area, and **Figure 6** visualizes the national and regional collaborations. From the map of national and regional collaborations of published literature in the field of osteosarcoma metabolism-related research shown in **Figure 6**, it can be observed that the field is radiating to other countries with China and the United States as the center. **Figures 6A-C** are the network of connections between countries or regions, the graph of author’s hotness over time, and the graph of author’s density, respectively. In addition, we have used the Bibliometrix package in R to perform another visual and clear geographic visualization of the field of osteosarcoma metabolism, as seen in **Figure 7**, which differs from **Figure 6** in that the patterns of the two diagrams allow for different presentations of the global collaborations in the field.

### Analysis of hot spots and possible future directions in the field of osteosarcoma metabolism research

Co-citation is defined as a network relationship in which at least one article appears to be cited for reference at the same time. One of these clusters is formed when a group of articles on similar topics are frequently cited together (30). Analysis of co-cited literature in the field of osteosarcoma metabolism. From the analysis of **Table 5** we found that “Osteosarcoma: Current Treatment and a Collaborative Pathway to Success” is the most cited article when it comes to published studies in the field of osteosarcoma metabolism. In addition, the top 10 cited articles in this field are shown in **Table 5**.

**Table 5:** Top 10 most cited references in common.

Rank	Cited reference	Citations	Total link strength
1	Isakoff ms, 2015, j clin oncol, v33, p3029, doi 10.1200/jco.2014.59.4895	37	34
2	Hanahan d, 2011, cell, v144, p646, doi 10.1016/j.cell.2011.02.013	35	34
3	Luetke a, 2014, cancer treat rev, v40, p523, doi 10.1016/j.ctrv.2013.11.006	34	22
4	Ottaviani g, 2009, cancer treat res, v152, p3, doi 10.1007/978-1-4419-0284-9_1	32	20
5	Bielack ss, 2002, j clin oncol, v20, p776, doi 10.1200/jco.20.3.776	30	21
6	Warburg o, 1956, science, v123, p309, doi 10.1126/science.123.3191.309	29	20
7	Mirabello l, 2009, cancer-am cancer soc, v115, p1531, doi 10.1002/cncr.24121	28	19
8	Heiden mgv, 2009, science, v324, p1029, doi 10.1126/science.1160809	26	25
9	Bradford mm, 1976, anal biochem, v72, p248, doi 10.1016/0003-2697(76)90527-3	24	1
10	Kansara m, 2014, nat rev cancer, v14, p722, doi 10.1038/nrc3838	23	20

### Analysis of keywords in the field of osteosarcoma metabolism

**Figure 8:**
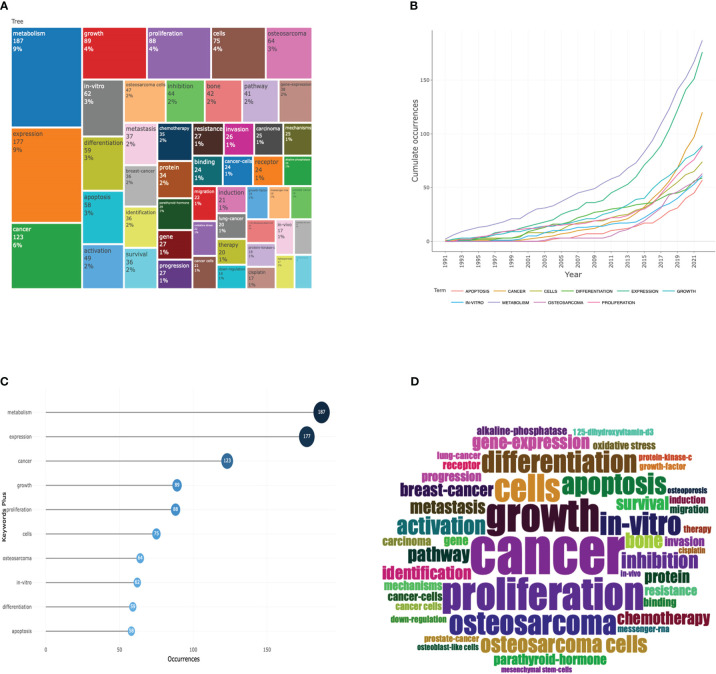
Analysis of keywords in the field of osteosarcoma metabolism research. **(A-D)** are the keyword percentage, keyword trend graph over time, keyword hotness and intensity, and keyword tree graph, respectively.

**Figure 9:**
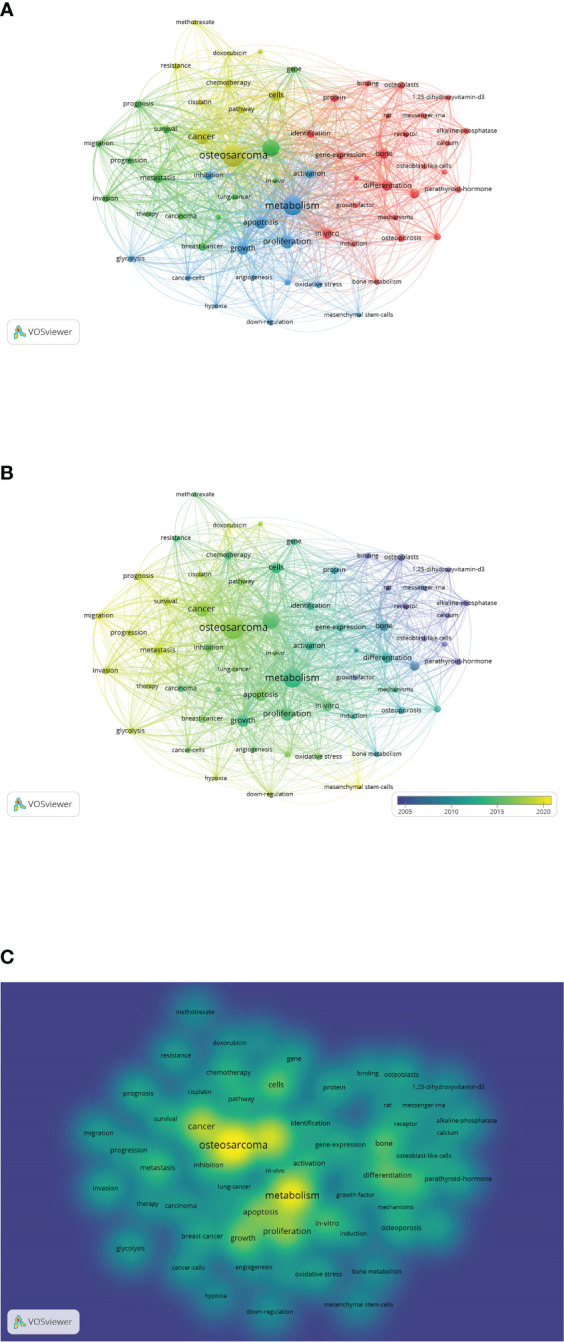
Collaborative network diagram of keywords in the field of osteosarcoma metabolism research. **(A-C)** are the connection network among keywords, the graph of keywords’ hotness over time, and the graph of keywords’ density, respectively.

The number and frequency of keyword occurrences in a given time frame is an important way to assess the current and future trends of a research field. **Figures 8A**, **Figure 8D** show the most frequently occurring keywords in the research field of osteosarcoma metabolism for the period 1990 to 2022, where the size can indicate their importance and frequency of use in the field. In addition, **Figure 9** shows the visualization of keywords using VOSviewer software, a network diagram that can clearly visualize the heat and trends of keywords. **Figure 8B** shows the results of the keywords in this field over the past 30 years with the change of years, from which we can find that the terms “metabolism” and “expression” have grown explosively, which also reflects the graph shows the current and possible future directions of the field. **Figure 8C** shows the top 10 keywords in this field. **Figures 8A-D** are the keyword percentage, keyword trend graph over time, keyword hotness and intensity, and keyword tree graph, respectively. **Figures 9A-C** are the connection network among keywords, the graph of keywords’ hotness over time, and the graph of keywords’ density, respectively.

### Analysis of the burgeoning word in the field of osteosarcoma metabolism research from 1990 to 2022

**Figure 10:**
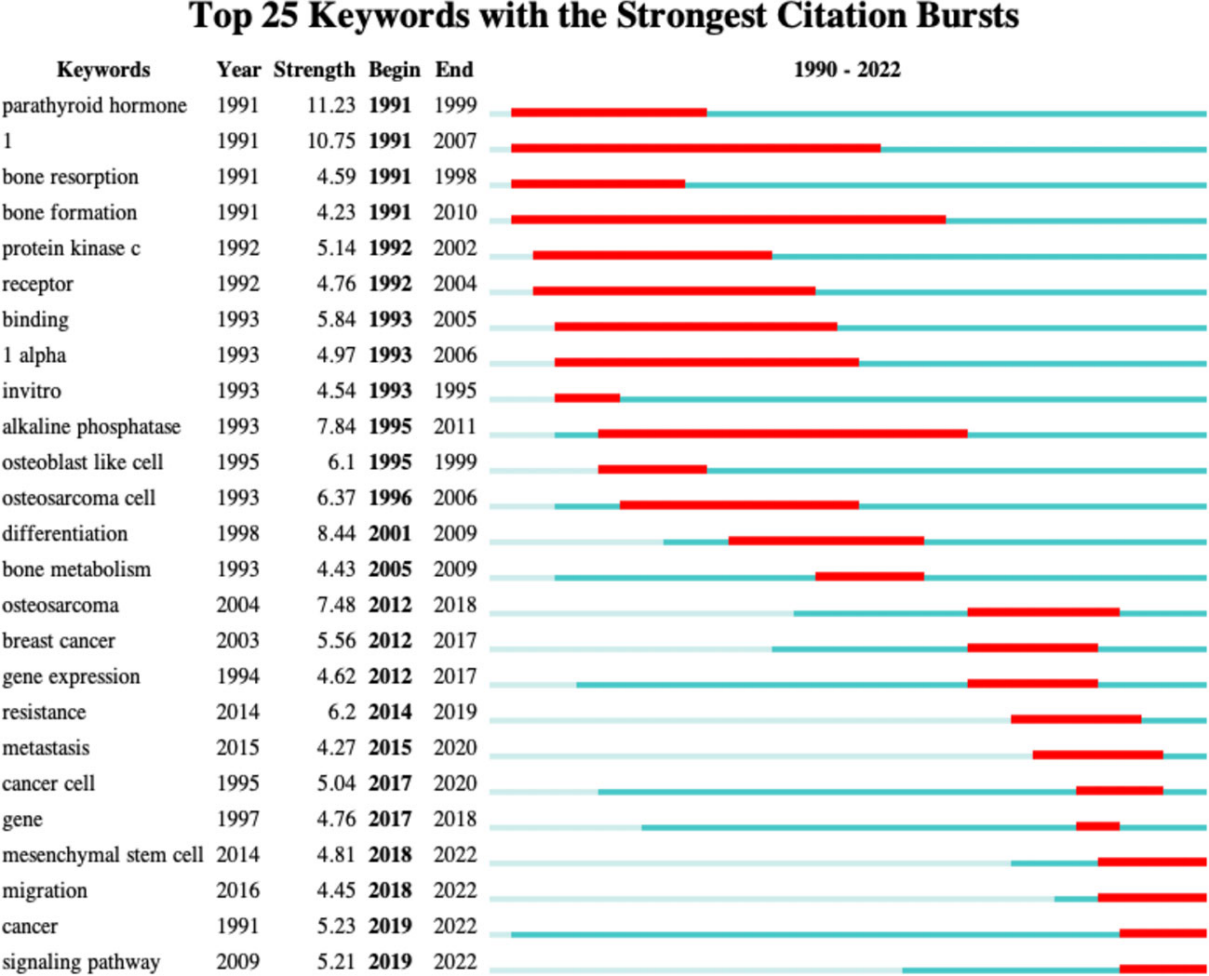
The top 25 highest intensity of keywords in the field of osteosarcoma metabolism research, i.e. outbreak words.

A unique feature of CiteSpace is that it can show the sudden explosion of terms in a certain research area at certain time periods to reflect the possible hot spots and trends at each time period. We used this software to analyze the words that broke out in the field of osteosarcoma metabolism, which is shown in **Figure 10**. The red color in the line following each outbreak term indicates its sudden outbreak during that time period.

## Discussion

We have done considerable research analysis work to deeply analyze and visualize the authors, country regions, and journals of articles published in the field of research related to osteosarcoma metabolism between January 1, 1990 and December 20, 2022. Based on this, we tried to show the current status of research in this field and possible future hotspots and trends, contributing to the development of the field and further value creation. We used Citespace, VOSviewer and other software to make a visual analysis of the last 30 years of research in this field, we found that there are 833 articles in this field, of which 5 of the top 10 published institutions are Chinese universities, but the most cited institution is the University of Bologna. China is the most cited country in the field, while the United States is the most cited overall and Canada is the number one cited on average.

In terms of authors, we found that in addition to the previously described, Baldini, nicola also studied the relationship between osteosarcoma cell secretion and metabolism and nanoparticles (31) and also Hall, ih mainly studied some elements of multiple drugs in the field of osteosarcoma metabolism studies, such as *in vitro* studies of osteosarcoma cells (32) and anti-tumor studies of compounds (33–35).

From the results of our analysis, China and the United States are the absolute centers of research in the field of osteosarcoma metabolism, with 470 publications from these two countries, accounting for about half of the global number of publications in this field.

The keywords found in the results are “metabolism”, “expression”, “cancer”, “osteosarcoma”, and “growth”. and “growth” are the words that appear very frequently. These words also indicate roughly the hottest research directions in the field between 1990 and 2022.

In addition, we analyzed the outbreak terms that have emerged in the field over the past 30 years, a method unique to Citespace software. It is a term that mainly reflects whether there are significant changes in a research field during a specific period of time, and can indicate hot spots and future trends for researchers. In the field of osteosarcoma metabolism research, we found that the main outbreak words in recent years are “cancer cell”, “mesenchymal stem cell”, “ migration”, “cancer”, and “signaling pathway”, which will mostly emerge and explode in 2017-2022. This can also predict that the research field in osteosarcoma metabolism may revolve around these hot spots in the coming years.

Our study is the first bibliometric analysis and visualization of this research area of osteosarcoma metabolism. Of course, our bibliometric’s have limitations inherent to this type of study. It is difficult to achieve simultaneous use of multiple databases in a bibliometric study, so we used only the WoS database, but considering that the WoS database is the most widely used, recognized, and covered database in bibliometrics (36, 37), the results generated reflect the overall trend.

## Conclusion

We conducted a bibliometric study and visualization of research in the field of osteosarcoma metabolism from 1990 to 2022 using several software such as VOSviewer and Citespace. From the results of our presentation, it is clear that metabolic research in the field of osteosarcoma is slowly becoming a hot direction in the field of osteosarcoma research with good prospects. baldini, nicola, Reddy, Gs, and Avnet, Sofia are the three authors with the highest number of publications in this field. China, USA and Japan are the three countries with the highest number of publications in this field. Shanghai Jiao Tong University, University of Bologna and Zhejiang University are the top 3 academic institutions in terms of number of publications. mesenchymal stem cell”, “migration”, “cancer”, and “signaling pathway” are the potential research hotspots in this field in recent years and in the future. This study is the first review of nearly three decades of research on relevant aspects of osteosarcoma metabolism through bibliometric analysis and provides a reference for future research.

## Data availability statement

The original contributions presented in the study are included in the article/supplementary material. Further inquiries can be directed to the corresponding author.

## Author contributions

ZS performed a bibliometric analysis and visualization study of articles in this field using several software. ZS wrote the manuscript. SB checked the manuscript several times. All authors contributed to the article and approved the submitted version.
